# Isolation of the Arawete and Asurini Indians keeps the tribes free from HTLV infection during 36 years of follow-up

**DOI:** 10.1186/s12977-019-0490-1

**Published:** 2019-10-22

**Authors:** Antonio C. R. Vallinoto, Mateus I. Otake, Paulo V. N. R. Sousa, Felipe T. Lopes, Eliene R. P. Sacuena, Maria A. F. Queiroz, Greice L. C. Costa, Marluísa O. G. Ishak, Izaura M. V. Cayres-Vallinoto, João F. Guerreiro, Ricardo Ishak

**Affiliations:** 1 Virus Laboratory, Institute of Biological Sciences, Federal University of Para, Rua Augusto Corrêa No.1, Belém, Para 66075-110 Brazil; 2 Human and Medical Genetics Laboratory, I Institute of Biological Sciences, Federal University of Para, Belém, Para Brazil

**Keywords:** HTLV-1/2, Indian tribe, Arawete, Asurini, Amazon region

## Abstract

Arawete and Asurini Indian tribes were revisited after a 36-year follow-up in search of HTLV infections. 46 persons (23 from each tribe) were tested for HTLV-1/2 antibodies and viral DNA. None were positive; this was probably because of their social/cultural isolation from neighboring tribes where HTLV-2c is hyperendemic.

Human T-lymphotropic virus 2, a member of the family *Retroviridae,* has been reported as a hyperendemic infection in several Amerindian tribes since 1992, particularly in the Brazilian Amazon region, among the Kayapó villages of the Jê linguistic group [1–3]. Furthermore, studies have shown evidence of HTLV-2 circulating among other Indians from different linguistic groups in the Amazon region of Brazil [3–6], in which breastfeeding and sexual intercourse are the main transmission routes [3, 5, 7, 8]. Phylogenetic and molecular analyses of the viral strain reported a new molecular subtype termed HTLV-2c, which is largely distributed in the Amazon region of Brazil [3, 4].

Since the early 1980s, native Indian tribes of the Amazon region of Brazil have been constantly receiving health support from our laboratories to monitor the spread of viruses and bacterial infections, particularly those transmitted by the sexual route. Since our initial large-scale testing [4], HTLV-1/2 have been routinely investigated to monitor their spread in both previously infected and virus-free villages. The present paper reports the maintenance of HTLV-free areas of infection among the Arawete (Igarapé Ipixuna-Médio Xingu, Para State, Brazil) and Asurini (Koatinemo-Médio Xingu, Para State, Brazil) groups belonging to the Tupí-Guarani linguistic group.

The Arawete and Asurini tribes were revisited in 2019, and again, the possibility of HTLV-1/2 emergence in their communities was monitored. The project was approved by the National Committee for Ethics in Research (CONEP), process 961.451/2015. Both visits received the agreement and consent of the communities through their leaders on behalf of the participants with formal written authorization, together with the National Indian Foundation (FUNAI), to offer health support and to investigate the presence of antibodies to infectious agents.

Table 1 describes the demographic information of forty-six subjects, 18 males and 28 females, with ages ranging from 5 to 85 years old, from the Arawete (n = 23) and Asurini (n = 23) tribes (Xingu region, State of Para) who were screened for anti-HTLV-1/2 antibodies by enzyme-linked immunosorbent assay (ELISA, Ortho Diagnostic, Raritan, NJ, USA). No positive or indeterminate reactions were observed. To avoid false negative results, such as those found among the Arara do Laranjal tribe [9], all the samples were submitted to a Strip Immunoblot Assay (Chiron*RIBA HTLV-I/II SIA, Johnson & Johnson Company, Raritan, NJ, USA) and a real-time polymerase chain reaction (qPCR) to the HTLV-2-*pol* gene, as previously described [8]. Immunoblot confirmed the absence of antibodies for HTLV-1/2, and qPCR confirmed the absence of HTLV-2 infection in the Arawete and Asurini tribes 36 years after their first investigation, suggesting that cultural and social isolation of these villages kept them free of the infection from other neighboring tribes where HTLV-2 is hyperendemic.

**Table 1:** Demographic data from the Asurini and Arawete tribes and their neighboring HTLV-2 infected Indian communities

Ethnicity	N	Male	Age range	Female	Age range	HTLV-2 (%)	Reference
Arawete	23	10	25 to 85	13	26 to 83	0	*
Asurini	23	8	5 to 72	15	17 to 63	0	*
Kayapó	200	86	0 to > 70	114	0 to > 70	33	3
Arara Laranjal	47	26	NI	21	NI	11.4	3
Parakanã	52	NI	NI	NI	NI	1.9	3
Kararaô	24	11	NI	11	NI	12.5	7
Xikrin	257	146	2 to 90	111	2 to 90	29	8

*NI* No information available

*Present study

Both Indian groups, Arawete (4°51′S and 52°21′W) and Asurini (4°12′S and 52°26′W), reside within reservations located in the State of Para, Brazil, and are surrounded by other communities, including the Kararaô (Jê linguistic group), the Arara do Laranjal (Karib), the Parakanã (Tupi), the Xikrin do Cateté (Jê) and several Kayapó villages (Jê) living in the same reservation (Fig. 1). It is important to mention that the prevalence of HTLV-2 ranged from 1.9 to 33% within these communities in our first visits (Table 1), and the most recent investigation that revisited three Xicrin villages found a continued high prevalence of infection [8]. Hyperendemicity of HTLV-2 among these communities is commonly sustained by sexual and mother-to-child (during pregnancy and perinatal breastfeeding) transmission [3–9]. Geographical proximity among these reservations was not an obstacle to the Asurini and Arawete villages in maintaining the cultural and social isolation during the years that prevented their interethnic mixing with neighboring Indian and non-Indian communities; their historical reports of ethnic conflicts [10] are important factors that have most likely prevented the virus from emerging among them.

**Fig. 1:**
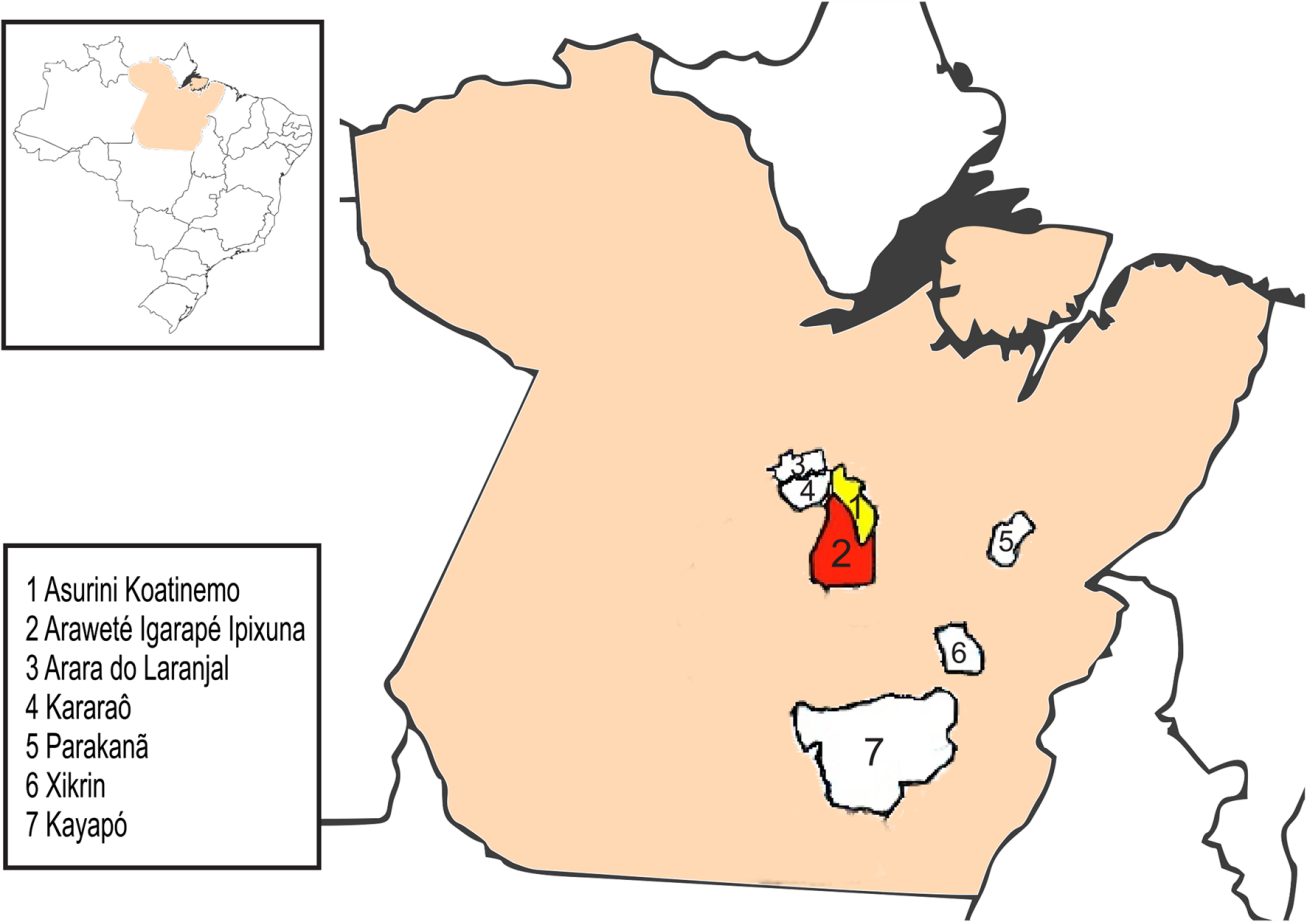
Geographical location of Asurini and Arawete reserves and their neighboring HTLV-2 infected Indian communities in the Para State, Brazil

The Indian populations of the Amazon region of Brazil are, to a great extent, epidemiologically closed or semiclosed communities with little or no interaction at all with other population groups, suggesting that the virus is an ancient infection among Indian populations of the Amazon region of Brazil [6]. The occurrence of HTLV-2 among distinct ethnicities is possibly associated with a typical founder effect [11], a usual demographic process that occurred during the formation of several Indian populations [12]. It is a common component of the formation of new communities during the fission of older and larger groups and new fusions to establish new communities. The founder effect reduces the presence of the virus from a stock population and, by chance, may select negative persons to establish a new smaller group. This is clearly evident when one considers the split of the Kararaô group, which was originally from the large Kayapó group of villages. Prevalence rates were down from a mean of 33% (Kayapó villages) to 12.5% (Kararaô). Infectious agents such as HTLV-2, which persistently infect the host, are maintained within familial clusters [3, 7, 13] and then efficiently spread within epidemiologically closed groups via sexual and vertical routes [3–9]. The Jê linguistic group within the Kayapó and the Xikrin reservations is an aggregate of villages with more than 2000 individuals, and both kept the virus despite the chance of village formations. The population stock that gave rise to the Arawete and Asurini (both Tupi linguistic groups) did not carry the virus, and this epidemiological situation continues to the present day.

The persons investigated were 5–85 years old, which means they were born before and after the first contact in the 1980s. The sample size, although apparently small, represents approximately 20–50% of the total inhabitants of the villages. The Asurini, following their initial contact in the 1970s, suffered a population decrease from 100 to 52 persons, mostly because of infectious diseases originally unknown to the community, and they were never able grow again due to their abortion and infanticide practices [14]. The Arawete have had constant conflicts with the Asurini and the Parakanã (also a Tupi linguistic group) since the 1970s, and the population is hardly more than 300 individuals in a single village by the Ipixuna *igarapé* on the right bank of the Middle Xingu [14, 15]. The Arawete maintain an ancient social habit of exchanging partners [15], which would impact the virus spread if HTLV-2 were present.

Ongoing health expeditions to Indian tribes for the last 40 years have proved successful from the viewpoints of providing health access and continuous epidemiological surveillance to prevent the spread of infectious agents including HTLV-2 among Indian communities and neighboring rural populations.

The investigation of HTLV-1/2 infection among the Arawete and Asurini Indian tribes over a follow-up of 36 years reinforces that social and cultural isolation of the villages, motivated by historical conflicts, kept them free of infection from other neighboring tribes where HTLV-2 is sometimes hyperendemic.

## Data Availability

The datasets used and/or analysed during the current study are available from the corresponding author on reasonable request.

## Abbreviations

HTLV: human T-lymphotropic virus
CONEP: National Committee for Ethics in Research
FUNAI: National Indian Foundation
ELISA: enzyme-linked immunosorbent assay
qPCR: real-time polymerase chain reaction
